# Supplementary material for: FERN – a Java framework for stochastic simulation and evaluation of reaction networks
Source: BMC Bioinformatics. 2008 Aug 29;9:356. doi: 10.1186/1471-2105-9-356 (PMC2553347; doi:10.1186/1471-2105-9-356)
Supplement: Additional file 1 — FERN distribution, Version 1.3. This archive contains the FERN source code and binaries as well as documentation and example models in FernML and SBML. [file 1471-2105-9-356-S1.zip › fern/doc/javadoc/fern/network/sbml/SBMLEventHandlerObserver.html]

SBMLEventHandlerObserver


---


|  |  |  |
| --- | --- | --- |
| **Overview** \| **Package** \| **Class** \| **Use** \| **Tree** \| **Deprecated** \| **Index** \| **Help** | |  |
| **PREV CLASS**   **NEXT CLASS** | **FRAMES**    **NO FRAMES**     **All Classes** |  |
| SUMMARY: NESTED \| FIELD \| CONSTR \| METHOD | DETAIL: FIELD \| CONSTR \| METHOD |  |


---


## fern.network.sbml Class SBMLEventHandlerObserver

```
java.lang.Object
  fern.simulation.observer.Observer
      fern.simulation.observer.TriggerObserver
          fern.network.sbml.SBMLEventHandlerObserver
```

---

``` public class SBMLEventHandlerObserver extends TriggerObserver ```

Observer which handles an event of a sbml model.

**Author:**
:   Florian Erhard

---

**Constructor Summary**

`SBMLEventHandlerObserver(Simulator sim,
SBMLNetwork net,
org.sbml.libsbml.Event event)`
  
          Creates the observer.


| **Method Summary** | |
| --- | --- |
| `void` | `activateReaction(int mu, double tau, Simulator.FireType fireType, int times)`             Gets called before a reaction fires. |
| `void` | `finished()`             Gets called when a simulation has finished, directly after the termination check. |
| `void` | `setSimulatorAsync(Simulator sim)` |
| `void` | `started()`             Gets called when the simulation has started after the initialization and before the termination condition is checked the first time. |
| `void` | `step()`             Gets called after each termination check and before `Simulator.performStep(fern.simulation.controller.SimulationController)` is called. |
| `void` | `theta(double theta)`             Gets called by simulators when a certain moment in time is reached. |
| `String` | `toString()` |
| `boolean` | `trigger()`             Is executed, whenever a event can trigger (before step and during a theta event). |

**Methods inherited from class fern.simulation.observer.Observer**

`applyLabelFormat, getLabel, getLabelFormat, getNumSimulations, getPrintWriter, getSimulator, getTheta, getTitlesCommand, print, setLabelFormat, setPrintWriter, setSimulator, setTheta`

**Methods inherited from class java.lang.Object**

`clone, equals, finalize, getClass, hashCode, notify, notifyAll, wait, wait, wait`

**Constructor Detail**

### SBMLEventHandlerObserver

```
public SBMLEventHandlerObserver(Simulator sim,
                                SBMLNetwork net,
                                org.sbml.libsbml.Event event)
```

:   Creates the observer.

    **Parameters:**: `sim` - the simulator: `net` - the sbml network: `event` - the event object of the sbml model


**Method Detail**

### activateReaction

```
public void activateReaction(int mu,
                             double tau,
                             Simulator.FireType fireType,
                             int times)
```

:   **Description copied from class: `Observer`**
:   Gets called before a reaction fires.

    :   **Specified by:**: `activateReaction` in class `Observer`
    :   **Parameters:**: `mu` - the reaction which is supposed to fire: `tau` - the time the reaction fires (at this time `Simulator.getTime()` does not necessarily yield the firing time): `fireType` - the type of the firing: `times` - TODO

---


### finished

```
public void finished()
```

:   **Description copied from class: `Observer`**
:   Gets called when a simulation has finished, directly after the termination check.

    :   **Specified by:**: `finished` in class `Observer`

---


### started

```
public void started()
```

:   **Description copied from class: `Observer`**
:   Gets called when the simulation has started after the initialization and before
    the termination condition is checked the first time.

    :   **Specified by:**: `started` in class `Observer`

---


### step

```
public void step()
```

:   **Description copied from class: `Observer`**
:   Gets called after each termination check and before `Simulator.performStep(fern.simulation.controller.SimulationController)`
    is called.

    :   **Specified by:**: `step` in class `Observer`

---


### trigger

```
public boolean trigger()
```

:   **Description copied from class: `TriggerObserver`**
:   Is executed, whenever a event can trigger (before step and during a theta event).

    :   **Specified by:**: `trigger` in class `TriggerObserver`
    :   **Returns:**: whether or not the trigger has fired

---


### theta

```
public void theta(double theta)
```

:   **Description copied from class: `Observer`**
:   Gets called by simulators when a certain moment in time is reached. This moment in
    time has to be registered by `Observer.getTheta()`

    :   **Specified by:**: `theta` in class `Observer`
    :   **Parameters:**: `theta` - moment in time

---


### setSimulatorAsync

```
public void setSimulatorAsync(Simulator sim)
```

---


### toString

```
public String toString()
```

:   **Overrides:**: `toString` in class `Object`


---


|  |  |  |
| --- | --- | --- |
| **Overview** \| **Package** \| **Class** \| **Use** \| **Tree** \| **Deprecated** \| **Index** \| **Help** | |  |
| **PREV CLASS**   **NEXT CLASS** | **FRAMES**    **NO FRAMES**     **All Classes** |  |
| SUMMARY: NESTED \| FIELD \| CONSTR \| METHOD | DETAIL: FIELD \| CONSTR \| METHOD |  |


---
